# A Patent Dip

**Published:** 1857-12

**Authors:** 


A "PATENT DIP," scientific American, literary wife, professional husband, a love of a baby as tidy as a lily and as sweet as a new-blown rose, cleanliness and health, labor saving, long nights of undisturbed sleep, all are concatenated in this triliteral monosylable "DIP." There is not a young family in the land to whom it may not be fraught with blessings.

It is the invention of a literary gentleman, driven thereto by a literary wife, learned in all things, except in the ability to keep her house and children in perfect neatness; and having to keep themselves, we may well judge it was a miserable failure. Thinking and studying hard all day, the exhausted brain would fall into blissful obliviousness as soon as the head touched the pillow, and would so remain until next morning—and so would the baby, with all its surroundings, imaginable and unimagined.

A true man is never discouraged, except by a demonstrable impossibility; so this gentleman determined that there ought to be a way, and what ought to be should be, and was, and he would find it out. Every morning intensified his convictions, and he had no rest. What cogitations, what solitary walks, and how long, what day-dreams and night realities, what hopes and what desperations there were, is not computable, *nor are the results*, in the way of cleanliness, health, and undisturbed repose.

Dr. REES, in an able monograph on infant mortality, asserts, that in this country nearly half of all who are born die in infancy, that is, under five years of age. We believe that three fourths of these untimely deaths result from *the want of proper attention to children*; and among the first of the items under this head is a want of personal cleanliness as to feet, hands, body, clothing, bedding, every thing.

The article for which letters patent have been obtained, according to the *Scientific American*, is a Diaper for Children, and is one of the most simple, ingenious, and desirable domestic articles to which our attention has been lately directed. The person of the child is kept dry, and in perfect cleanliness, odors are prevented, and there is no necessity for a change during the longest night, one ablution only is required, and it is ready for use again in a few minutes. So contrived, too, that all the dangers are obviated which result from children sitting on cold or damp seats, as well as the inconveniences in travelling.

That six thousand years should have passed, and some mind has not sooner brought its energies to bear for the abatement of a nuisance so universal, is noticeable. Yet the thing is claimed to be done; and if so, we trust that the pecuniary reward will be commensurate with the satisfaction experienced by fruitful parents for generations to come.

The saving in washing alone, for three months, in a city, is claimed to be enough to purchase a supply of the article sufficient during the rearing of an ordinary family; and if actual experiment certifies these claims, the old article will fall into disuse, not to be resuscitated.

